# Non- Pharmacological Approaches on the Improvement of Sleep Disturbances in Patients with Autism Spectrum Disorder (ASD)

**DOI:** 10.22037/ijcn.v15i2.25539

**Published:** 2021

**Authors:** Faraz TAYYAR IRAVANLOU, Mohammad SOLTANI, Fatemeh ALSADAT RAHNEMAEI, Fatemeh ABDI, Mahnaz ILKHANI

**Affiliations:** 1 Psychiatric Nursing Student, Student Research Committee, School of Nursing and Midwifery, Shahid Beheshti University of Medical Sciences, Tehran, Iran; 2 Student Research Committee, Nursing and Midwifery Faculty, Shahid Beheshti University of Medical Sciences, Tehran, Iran.; 3 Social Determinants of Health Research Center, Alborz University of Medical Sciences, Karaj, Iran; 4 School of Nursing and Midwifery,Shahid Beheshti University of Medical Sciences, Tehran, Iran

**Keywords:** Autism, sleep disturbance, Physical Activity

## Abstract

**Abstract:**

Autism is a type of neurodegenerative disorder, caused by genetic and environmental factors. Children with autism spectrum disorder (ASD) have symptoms of attention deficit and behavioral problems. Child’s sleep pattern has a significant effect on mood. Sleep problems are more common in children with ASD. The current study aimed to investigate the effect of non-pharmacological approaches on the improvement of sleep disturbances in patients with ASD.

**Materials & Methods:**

We systematically searched PubMed, EMBASE, Web of Science, Scopus, and Science Direct to identify relevant articles published from January 2009 to May 2019. All original articles from observational and interventional studies were reviewed. The CONSORT Statement and Strengthening the Reporting of Observational Studies in Epidemiology(STROBE) checklist were used to assess the quality of selected papers.

**Results:**

Reviewing 18 eligible articles according to the CONSORT checklist(for interventional studies) and STROBE (for descriptive studies) demonstrated that behavioral interventions, such as cognitivebehavioral therapy, bedtime fading with response cost, and functional behavioral assessment, physical activity such as aerobic exercise, swimming, and aquatic exercise and weighted blankets can improve sleep disturbances.

**Conclusion:**

Restlessness, tantrums, increased stereotypic activities, and reduced

learning level and speaking power in children with autism were. caused by poor sleep quality and sleep deficiency, which may affect other dimensions of life. Non- pharmacological approaches to sleep disturbances could improve both sleep quality and quality of life of children with ASD with no adverse effect.

## Introduction

Autism is characterized by difficulties in social interactions and communications and a tendency to engage in repetitive behaviors (1). Several genetic and environmental factors contribute to this neurodevelopmental disorder (2). Autism Spectrum Disorder (ASD) is most likely to be affected by social-cognitive impairments and has a distinct brain structure and function (3). Children with ASD present symptoms of deficits in attention, social skills, and behavior (4). New diagnostic criteria for ASD in the Diagnostic and Statistical Manual of Mental Disorders, Fifth Edition (DSM-V) have focused on two main domains: (1) social communication disorder, and (2) restricted, repetitive patterns of behavior or interests. Diagnosis of ASD is based on difficulties in three areas of social communication and two areas of restricted, repetitive patterns of behavior or interests. During the past decades the prevalence of ASD has increased steadily. According to the latest data available, the prevalence of ASD among children is 1 in 36. Besides, it's reported that the mean prevalence of ASD in Asia, Europe, and North America is around 5% (5). Also, boys are four times more likely to develop ASD than girls. More than 75% of ASD cases are linked with other neuropsychiatric disorders, which may increase the complicacy of diagnosing the disease (6). ASD usually appears in a child's first 3 years of life (mostly 18 to 24 months); therefore, the definitive diagnosis is also around this period (7). The increased incidence of ASD indicates the need for efficient, sensitive, and targeted tools to assess the disease (8). As ASD is a spectrum disorder, it can be treated using various therapeutic options, including pharmaceutical and non-pharmacological approaches for managing autism-related problems. The appropriate therapeutic option should be chosen based on the child's needs, with special attention to reducing symptoms, enhancing learning, and promoting development. Currently available approaches to treat ASD contain pharmaceutical treatments, complementary therapies, and behavioral and communication treatments (9, 10). Most of the ASD children (38-44%) suffer from sleep disturbances. Other common sleep disorders include sleep latency, restless sleep, and waking up frequently during the night (11). Therefore, there are many therapeutic approaches to improve sleep disturbances.

Children with autism need treatments with high flexibility. Cognitive Behavior Therapy (CBT) is a commonly used targeted strategy to adapt patients with autism to adaptive patterns of thinking and behavior, that results in positive changes in feelings and reduced functional impairment (12). The CBT is used as a therapeutic approach to behavioral problems in autism, such as aggression, sleep problems, and other disorders. CBT programs emphasize social accountability and focusing on helping patients to develop cognitive abilities, which in turn result in improved social interaction (1). CBT is a psychotherapy intended to help people to understand the interconnectivity between their thoughts, behaviors, and feelings. This approach is also useful for finding new ways to think, cope, and respond to anxiety (13). It's well-documented that physical activity has positive impacts on the sleep quality of ASD children. For example, according to the evidence, jogging, strength exercises, and regular physical activity can improve the sleep quality of those with ASD (14). 

Other behavioral interventions for sleep problems of ASD children include regulating nighttime sleep, scheduled awakening, sleep restriction, parental training program, and bedtime fading with and without response cost (15). Weighted blankets also influence sleep quality (16). There are systematic reviews, with explicit and explicit goals, which have summarized the reported outcomes as well as the best form of evidence for judgment (17)(16). Systematic reviews can summarize reported outcomes by several studies and provide the highest form of evidence for scientific judgment (17). According to the best knowledge of the authors, no systematic review has investigated the effects of non-pharmacological approaches on improving sleep disturbances in patients with autism. Therefore, the results of this study can help make decisions about the prescription of alternative therapies, instead of drug therapy, with lower risks. The current study aimed to investigate the effect of non-pharmacological approaches on improving sleep disturbances in patients with ASD.

## Materials & Methods

***Search strategy***

The present study is reported based on the Preferred Reporting Items for Systematic Reviews and Meta-Analyses (PRISMA) guidelines. We systematically searched MEDLINE, Web of Sciences, PubMed, Scopus, Google Scholar, and ProQuest to identify relevant articles published.

**Table 1:** Search strategy

	Search term
#1	'Autism Spectrum Disorder' [tiab], OR 'Autistic' [tiab], OR 'Autistic Disorder' [tiab], OR 'Autism Syndrome' [tiab], 'Asperger Syndrome'[tiab]
#2	'Sleep disturbance' [tiab] OR 'Sleep problem' [tiab], OR 'sleep disorder' [tiab], OR 'insomnia' [tiab] 'Dyssomnias' [tiab], OR 'Sleep Deprivation' [tiab], OR 'Sleep Wake Disorders' [tiab], OR 'sleep difficulty' [tiab]
#3	' Cognitive Behavior Therapy (CBT)' [tiab], OR 'Physical Activity' [tiab], OR 'Behavioral Intervention'[tiab], OR 'Behavior Control'[tiab], OR 'Exercise'[tiab], OR 'Swimming Intervention'[tiab], OR 'weighted blankets'[tiab]
#1 AND #2	
#1 AND #3	
#1 AND #2 AND #3	

***Inclusion and exclusion criteria***

The inclusion criteria were as follows: (1) All Persian and English language articles (observational and interventional studies) published between January 2009 and May 2019, and (2) Studies which have investigated the effects of non-pharmacological approaches on improving sleep disturbances in patients with ASD.

The exclusion criteria were as follows: the lack of access to full-text articles; language other than Persian or English; protocol studies; case studies; brief reports; and only investigating parents and/or caregivers of individuals with ASD, as well as research performed on pharmacological and non-pharmacological treatments. 

***Study selection***

**Figure 1:**
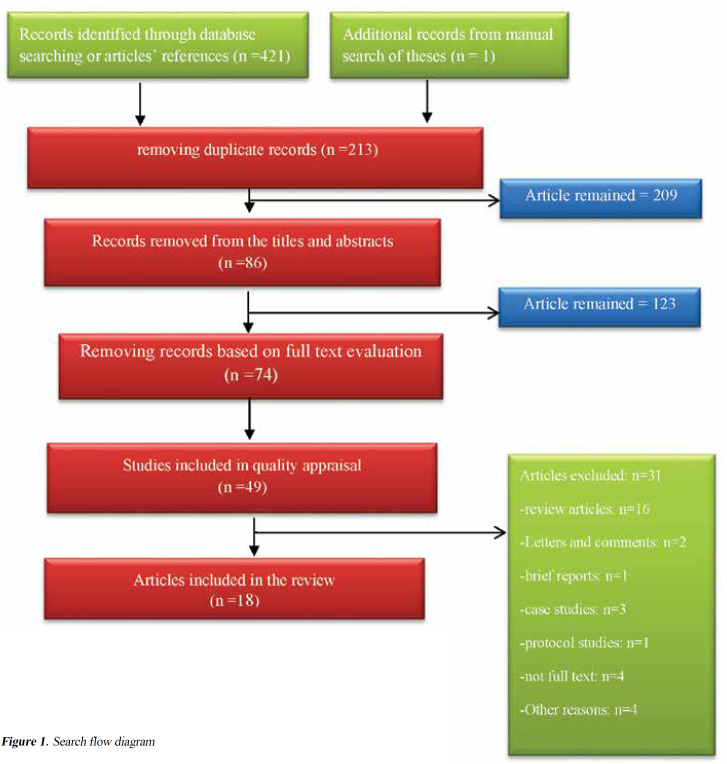
Search flow diagram

Initially, 422 studies were found. The eligibility of articles was independently evaluated by two researchers, and disagreements were resolved by consensus. In the first stage, 213 articles were excluded due to being irrelevant or duplicated. After reviewing the titles and abstracts of the remaining articles, 86 were excluded. After evaluating full texts, 74 (out of the remaining 123 articles) were excluded due to not matching inclusion criteria. Hence, in total 18 articles were reviewed (Figure 1).

***Quality assessment***

The CONSORT (Consolidated Standards of Reporting Trials) and STROBE statements were used to evaluate the quality of studies. The CONSORT Statement is a 25-item checklist, which is focused on reporting how the trial was designed, analyzed, and interpreted. STROBE Statement is an authoritative tool consists of a 22-item checklist, which is focused on reporting or evaluating different sections of observational studies (18, 19). 

***Data extraction***

Two authors independently evaluated the articles. In case of disagreement, a consensus was reached through discussion. The first author name, publication year, country, study design, sample size, age, quality assessment, and results were extracted and entered the analysis.

## Results

The search process used in the present study is presented in Figure 1. First, the titles and abstracts of the articles were reviewed. The full texts of the articles which had inclusion criteria were reviewed. Finally, 18 eligible articles with CONSORT scores between 16 to 24 (for interventional studies) or STROBE scores between 17 to 20 (for Descriptive studies) were included. Scoring the clinical trials and randomized clinical trials (RCTs) has been conducted in several different countries, such as the United States (11), Canada (1), Australia (2), New Zealand (1), Switzerland (1), Sweden (1), and Italy (1). Of 15 identified clinical trials, 6 were RCTs. Also, three observational, cohort, and cross-sectional studies were included. Overall, 1867 children and adolescents were investigated in these studies. Sleep problems included sleep latency, difficulty with falling asleep, insomnia, excessive fatigue, walking and talking during sleep, sleeping with light, and needing an individual to sleep. Thirteen studies that investigated behavior therapy assessments (e.g. CBT, bedtime fading with response cost and/ or functional behavioral), either individually or group, showed that sleep disturbance was significantly improved. Also, four studies which were investigated physical activity, such as swimming, aerobic exercises, and aquatic exercise in the form of organized programs indicated that the sleep quality of ASD patients was significantly improved. A study was also conducted on the use of weighted blankets.

**Table 2:** Results of systematic review of studies

Author (year)	Country	Study design	Sample size	Age (years)	Quality assessment(score)	Results
Tse (20) (2019)	USA	RCT	N=40 males: 32 female: 8	8–12	*23	Physical activity in children with ASD improved sleep quality and inhibition control
Sanberg(15) (2018)	USA	Clinical trial	N=3 males: 2 female: 1	4 - 8	*24	- BFRC (Bedtime Fading with Response Cost) as a parent-delivered intervention was associated with improved sleep onset latency, bedtime resistance, and disruptive sleep-related behaviors.
McLay (21) (2018)	New Zealand	Clinical trial	N=7 Males: 5 Females: 2	2 - 5	*19	-FBA (functional behavioral assessment) can inform interventions and the effectiveness of behavioral interventions for sleep problems in children with ASD.
Abel (22) (2018)	USA	Clinical trial	N = 39 Males: 33 Females: 6	2 - 10	*22	- Poor sleep was associated with higher rates of repetitive behavior, negative consequences, and a composite of overall challenging behaviors.
Kurz (9) )2018(	Austria	observational	N=9 all male	4-10	**17	- Significant changes were observed in irritability, lethargy, and hyperactivity after 12 months of CBT
Lawson (23) (2017)	USA	Clinical trial	N=10 Males: - Females: -	5-12	*18	After administering the intervention, sleep disturbance was decreased and sleep behavior was improved.
Benson (24) (2017)	Canada	Cross sectional	N= 15 Males: 11 Females: 4	18-35	20٭*	-Due to the low levels of physical activity in individuals with ASD, those with higher levels of physical activity were more likely to feel drowsy, fall asleep early, and wake up early and consequently they experienced improved subjective sleep quality and behavior and personality characteristics
Peterman (25) )2016(	USA	Clinical trial	N=69 male:34 Female:35	7-17	*24	-sleep-related problems were improved after CBT
Oriel (26) (2016)	USA	Clinical trial	N=8 Males: 5 Females: 3	6 - 11	*20	- After the intervention, all participants showed significant increases in sleep duration, and the number of their awakenings during the night was reduced, and they also fell asleep faster.
Gee (16) (2016)	USA	Clinical trial(ABA single-subject design)	N=2 Males: - Females: -	4-5	*16	-Using weighted blankets was associated with improved sleep quality in times of falling asleep, frequency of awakenings during the night, nighttime sleep duration, and morning behavior.
Brand (14) (2015)	Switzerland	Clinical trial	N=10 Females: 5 Male: 5	7 - 13	*22	-Aerobic exercise training and MS training could decrease sleep disturbance.
Papadopoulos (27) (2015)	Australia	RCT	N = 61 males: 54 females: 7	5 - 13	*24	- After face-to-face counseling and telephone follow up, sleep problems were improved
Hesselmark (28) (2014)	Sweden	RCT	N=68 Male: 41 Female:27	18≤	*24	- After providing the intervention, both sleep quality and the quality of life of the participants were improved.
Nadeau (29) (2014)	USA	Clinical trial	N= 102 males: 81 Female:21	7–16	*22	- Those who received CBT showed a significant reduction in sleep-related problems from beginning treatment to post-treatment.
Storch (30) (2014)	USA	RCT	N=31 Males: 25 Females: 6	11-16	*23	- Sleep tremor disorder was more improved in the CBT group than the usual treatment.
Sikora (31) (2012)	USA & Canada.	cohort	1193 N= Male:1014 Female: 179	4-10	**20	-There is a direct association between sleep and behavior problems, so that children with greater sleep disturbance experience higher levels of behavior disturbance.
Cortesi (32) (2012)	Italy	RCT	N=160 Males:131 Female:29	4-10	*22	- Combining CBT and CR-melatonin increases the improvement.
Drahota (33) (2010)	USA	RCT	N= 40 Males: 12 Female: 28	7–11	*23	- CBT increased the independence and daily living skills of ASD children.

RCT: Randomized Controlled Trial, CBT: Cognitive Behavioral Therapy, CR-melatonin: Controlled‐release melatonin, ASD: Autism Spectrum Disorder, PA: Physical activity

٭:by CONSORT

٭٭:by STROBE

## Discussion

This study demonstrated that behavioral therapy and physical activity, as non-pharmacological approaches and effective therapies, not only could improve sleep disturbances in individuals with ASD, but also no side effect was observed. Besides, administering these approaches was associated with improved quality of life of ASD patients.

Wood et al. (2015) conducted a study in the United States to investigate the effect of CBT in adolescents with autism and demonstrated that, based on Clinical Global Impression–Improvement (CGI-I), those who received this treatment presented a better improvement rate than those in the control group (79% versus 28.6%, respectively). The CBT model challenges irrational beliefs and behavioral supports provided by caregivers. Besides, it provides a basis for specialized treatments for autism (34). In a RCT conducted in Sweden by Hesselmark et al. (2014), titled “Group cognitive-behavioral therapy and group recreational activity for adults with autism spectrum disorders”, 68 psychiatric patients with ASD are classified by age and then assigned to one of the two treatment options. The authors reported no improvement in psychiatric symptoms, but participants in the CBT group have rated themselves as more generally improved and stated that they were more improved regarding the expression of needs and understanding difficulties. Both interventions appear to be promising therapeutic options for adults with ASD (35).

In the United States, Locke et al. (2014) carried out a study on 196 students from 47 kindergarten-through-second-grade autism support. In a year-long behavioral intervention study, the association between gains in cognitive ability and social functioning in children with autism was investigated. The findings of this study suggested that there is a basic need for cost-effectiveness, valid environment, and suitable psychometric measures for wide-scale use in socialization. Observed social gains were not commensurate with gains in cognitive ability, indicating the need for both interventions that are directly targeted to social functioning and relevant field measures for social functioning (36). Eack et al. (2013) conducted a study titled “Cognitive enhancement therapy for adults with autism spectrum disorder: results of an 18-month feasibility study”. in this study, satisfaction and acceptance levels of treatment were measured by Client Satisfaction Questionnaire-8 (CSQ-8). The results revealed that CET is a feasible, acceptable, and potentially effective intervention for remediating the social and non-social cognitive impairments in adults with autism. It worth noting that this study was conducted on a small sample size that was appropriate for assessing the feasibility of this measure, but caution should be taken when generalizing the results (37).

In addition to the above-mentioned results, they reported that sleep disturbances in individuals with autism who received CBT were also improved, and sleep duration, sleep quality, frequency of night awakenings, restlessness, and falling into sleep were improved (9, 25, 29, 32). To investigate other behavioral interventions in individuals with autism, Sanberg et al. (2018) examined the effect of bedtime fading with response cost on sleep disturbances in children with autism and reported that BFRC was effective in improving the sleep problems (15). McLay et al. (2018) investigated the effect of functional behavioral assessment on the treatment of sleep problems in ASD children; they also clearly demonstrated sleep problems were improved at the end of the intervention (21).

Children with low levels of physical activity are at increased risk of obesity and for children with autism this risk increases by 40% (38). Activity levels were significantly related to the age of children, as Wachob et al. (2015) demonstrated that older children were less likely to have high levels of physical activity because complex motor activities were more difficult to perform with increasing age, making it more difficult to do physical activities. Also, numerous studies have shown that going to school reduces the physical activity of ASD children, because they engage in academic school activities and have less time to do physical activities (39). Therefore, we observed a positive association between the daily physical activities of children with autism and their sleep quality at night. In particular, more active children acquired both good physical health and a regular sleep pattern throughout the night (40, 41).

In this regard, several studies have investigated the effects of different forms of physical activity on improving sleep disturbances in individuals with ASD. Brand et al. (2015) examined the effects of aerobic exercise training and MS training on improving sleep disturbances following a 3-week intervention. At the end of the intervention, they observed that all symptoms related to sleep disturbances were improved (14). In their studies, Lawson et al. (2017) investigated the effects of swimming intervention for 8 weeks on sleep disturbances of children with autism and observed that sleep disturbances were improved on the basis of specific characteristics of each child (23). Kathryn et al. (2016) also investigated the effects of four weeks of aquatic exercise on the sleep behaviors of children with autism, and found that these children fell asleep faster, awakened less at night, and their sleep duration was increased following the intervention (26). Feel-based intervention is another approach that was investigated in the present study. Gee et al. (2016) explored the efficacy of the weighted blanket intervention on two children with ASD using ABA design, and observed that the time to fall asleep, the number of nighttime awakenings, nighttime sleep duration, and morning behavior were significantly improved (24).

As mentioned before, Child's sleep pattern has a significant effect on mood. Sleep problems are more common in ASD children. In this regard, non-pharmacological treatments appear to be effective and helpful in improving sleep‐onset latency, staying awake, early awakening, sleep disruption, and decreased need for sleep. Therefore, further studies are needed to investigate the effect of CBT on the improvement of sleep in children with autism.

## Conclusion

This study demonstrated that the severity of autism spectrum disorder or ASD may range from mild to severe, and the disease is characterized by mental retardation, and aggression and hyperactivity. Insomnia and dyssomnia are common sleep problems in children with ASD, which increase their restlessness, tantrum, and stereotypic behaviors, and have a direct impact on their concentration, level of learning, and even speaking. Although the exact cause of sleep problems in these children is not clear, but some studies have mentioned physical, psychological, and environmental factors as the main causes. Physical activity, behavioral interventions, and feel-based interventions, as non-pharmacological approaches, can improve sleep quality in children with ASD and, ultimately, their quality of life.

## Limitations

Our literature review revealed that very few studies have investigated the effects of non-pharmacological approaches on improving the health status of ASD children. Besides, many of these studies were conducted on parents and caregivers of children with autism. Hence, further studies with larger sample sizes are needed. Another important limitation of the present study was the lack of access to the full texts of some articles.
